# Oral Lesions in Neonates

**DOI:** 10.5005/jp-journals-10005-1349

**Published:** 2016-06-15

**Authors:** Shankargouda Patil, Roopa S Rao, Barnali Majumdar, Mohammed Jafer, Mahesh Maralingannavar, Anil Sukumaran

**Affiliations:** 1 Associate Professor, Department of Maxillofacial Surgery and Diagnostic Sciences, Division of Oral Pathology, College of Dentistry Jazan University, Jazan, Kingdom of Saudi Arabia; 2 Professor and Head, Department of Oral Pathology and Microbiology, Faculty of Dental Sciences, MS Ramaiah University of Applied Sciences Bengaluru, Karnataka, India; 3 Postgraduate Student, Department of Oral Pathology and Microbiology, Faculty of Dental Sciences, MS Ramaiah University of Applied Sciences Bengaluru, Karnataka, India; 4 Lecturer and Head, Department of Preventive Dental Sciences, College of Dentistry, Jazan University, Jazan, Kingdom of Saudi Arabia; 5 Associate Professor, Department of Maxillofacial Surgery and Diagnostic Sciences, Division of Oral Pathology, College of Dentistry Jazan University, Jazan, Kingdom of Saudi Arabia; 6 Professor, Department of Preventive Dental Sciences, College of Dentistry, Jazan University, Jazan, Kingdom of Saudi Arabia

**Keywords:** Congenital, Dental, Neonates, Neoplasms, Newborns, Oral lesions.

## Abstract

Oral lesions in neonates represent a wide range of diseases often creating apprehension and anxiety among parents. Early examination and prompt diagnosis can aid in prudent management and serve as baseline against the future course of the disease. The present review aims to enlist and describe the diagnostic features of commonly encountered oral lesions in neonates.

**How to cite this article:** Patil S, Rao RS, Majumdar B, Jafer M, Maralingannavar M, Sukumaran A. Oral Lesions in Neonates. Int J Clin Pediatr Dent 2016;9(2):131-138.

## INTRODUCTION

The diseases of the oral cavity comprise an important arena of the pediatric specialty, yet many are misdiagnosed or left untreated due to lack of resources and parental education. Although the lesions are usually confined to the oral cavity, they might provide certain clues to the underlying more serious systemic conditions. A broad spectrum of diseases manifest with oral features in the neonates (Flow Chart 1 and Table 1), majority being asymptomatic and benign, commonly resolve without any intervention. However, a thorough clinical examination and knowledge of the various lesions is essential for precise diagnosis, management, as well as parental counseling.

## CYSTS

### Gingival/Dental Lamina Cyst of Neonates

The gingival cysts are frequently observed in newborns (13.8%) with no gender predilection.^1^ They are postulated to arise from the dental lamina. They appear as small, multiple, nodular, and white to creamish lesions on the crests of the maxillary and mandibular dental ridges. Histopathology reveals they are keratin-filled true cysts. Treatment is not indicated as they self-resolute.^2,3^

### Epstein Pearls

They are nonodontogenic, keratin-filled cysts with prevalence of 35.2% with no gender predilection.^1,2^ They are apparently entrapped epithelial remnants. These are clinically asymptomatic and appear as nodules in the mid-palatal raphe region along the line of fusion. Treatment is not indicated.^2,3^

### Bohn’s Nodules

They are keratin-filled cysts with prevalence of 47.4% with no gender predilection.^1,2^ They are apparently derivatives of palatal salivary gland structures. They clinically appear as numerous nodules along the junction of the hard and soft palate. Treatment is not indicated.^2,3^

### Eruption Cyst

Eruption cyst (EC) in neonates is a rarity. Clark et al have reported six cases of EC in neonates, while Bodner et al have reported two cases.^4^ Their origin may be from degenerative cystic changes in the reduced enamel epithelium or from the remnants of the dental lamina.^3,4^ The patho-genesis involves impediment of eruption by overlying dense fibrotic mucosa.^2^ These clinically present as bluish, dome-shaped, translucent, compressible swelling within the mucosa, overlying the erupting tooth. Diagnosis is aided by fine needle aspiration biopsy (FNAB). Treatment includes marsupialization or surgical extraction.^3,4^

### Epidermoid and Dermoid Cysts

They are rare benign developmental disorders with an incidence of 7% in the head and neck region. Approximately 30 cases have been reported in neonates.^5^ Common oral locations include floor of the mouth and the submental region (23.3%).^6^ These are clinically asymptomatic, slow growing cysts and diagnosed when enlarged. It poses respiratory distress and feeding difficulty in neonates. Diagnosis is based on prenatal/natal ultrasonography (USG), magnetic resonance imaging (MRI)/computed tomography (CT), FNAB, and histopathology, ruling out ranula, dermoid cyst, teratoma, heterotopic gastrointestinal cyst, duplication foregut cyst, and lymphatic malformation. Histologically, an epidermoid cyst is lined by only epidermis and a dermoid cyst shows presence of adnexal glands in addition. Treatment includes surgical enucle-ation. Recurrence is rare.^5^

**Flow Chart 1:**
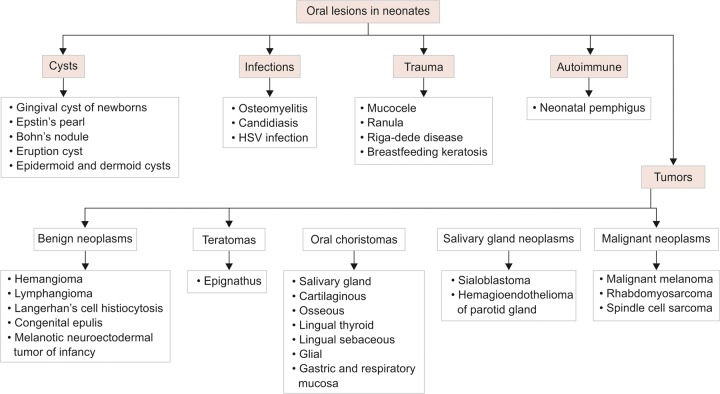
Working classification of neonatal oral lesions

**Table 1:** **Table 1:** Summary of oral manifestations of neonatal lesions

*Lesion*				*Location (oral cavity)*		*Oral manifestations*	
Cysts		Gingival and dental lamina cyst of newborn Epstein pearls		Crests of maxillary and mandibular dental ridges Mid-palatal raphe region along the line of fusion		Small, multiple, nodular and white to creamish lesions Small nodules	
		Bohn’s nodule Eruption cysts		Junction of the hard and soft palate A/w erupting teeth		Numerous nodules Bluish, dome-shaped, translucent, compressible swelling	
		Epidermoid and dermoid cysts		Floor of the mouth, submental region		Asymptomatic, slow-growing cysts	
Infections		Osteomyelitis Herpes simplex virus infection		Maxilla Mucous membrane		Edema and redness of cheek Vesicular eruptions in single unit or in clusters, which often ulcerate	
		Candidiasis		Mucous membrane		White plaques	
Trauma		Mucocele		Lower lip		Bluish, translucent, and fluctuant swelling	
		Ranula		Floor of the mouth		Similar to mucocele	
		Riga-Fede disease		Tongue (ventral surface)		Ulcerations, unifocal/multifocal, occasionally painful	
		Breastfeeding keratosis		Lower lip		White nonscrapable keratotic plaque	
Autoimmune		Neonatal pemphigus		Mucous membrane		Multiple ulcerations	
Benign neoplasms		Hemangioma		Lip, buccal mucosa, tongue, palate, uvula		Rapidly growing macule	
		Lymphangioma		Anterior two-thirds of tongue and submandibular and parotid area		Macroglossia, sialorrhea, dysphagia, ulcerations, deformity of jaws, and difficulty in speech, feeding, and mastication	
		Langerhans cell histiocytosis X		Jaws (mandible)		Petechiae, lytic bone lesions, pain and swelling of gingiva	
		Congenital epulis		Maxilla (alveolar ridge near the canine region)		Lobular or ovoid, sessile or pedunculated swelling	
		Melanotic neuroectodermal tumor of infancy		Tongue, palate, buccal mucosa, floor of the mouth		Painless, pigmented, nonulcerative, expansile, rapidly growing mass	
Teratomas		Epignathus		Hard palate, mandible		Unidirectional growth protruding through the oral cavity	
Oral choristomas		Glial, salivary gland, cartilaginous, osseous, etc.		Tongue, floor of mouth, pharynx, hypopharynx		Asymptomatic, large masses	
Salivary gland neoplasms		Sialoblastoma Hemangioendothelioma of salivary gland		Parotid, submandibular gland Parotid gland		Swelling, facial nerve palsy Multiple, rapidly growing mass	

## INFECTIONS

### Neonatal Osteomyelitis of Maxilla

It is a relatively rare infection with high mortality rate, seen in the neonatal period. The incidence is estimated to be 1 to 7 per 1,000 hospital admissions with a predilection for males (1.6:1) and preterm newborns. Common risk factors include iatrogenic, catheterization, prolonged hospital-ization, parenteral nutrition status, ventilatory support, and nosocomial infections.^7^ The causative organisms include *Staphylococcus aureus* (most common), group B Streptococcus *(Streptococcus agalactiae),* and Gram-negative organisms *(Escherichia coli* and *Klebsiella pneumonia).^7,8^* The involvement of maxilla is approximately 4% and the initial clinical presentations include acute onset of fever followed by edema and redness of cheek, swelling of eyelids with conjunctivitis, and unilateral nasal discharge.^9^ The features of chronic osteomyelitis is seldom manifested in new-borns. Diagnosis is based on positive blood cultures and tests (erythrocyte sedimentation rate, C-reactive protein, leukocyte count).^8^ Treatment includes empirical regimen of antimicrobial drugs along with or without surgical intervention. Prognosis is poor and morbidity is high.^7^

### Neonatal Herpes Simplex Virus Infection

It was first reported by Hass and Batignani in the mid-1930s. The incidence ranges from 1 in 3,000 to 20,000 live births. The orolabial lesions are caused by herpes simplex virus (HSV)1, while genital lesions are by both HSV1 and HSV2. The transmission occurs during the time of parturition and is facilitated by the status of maternal antibody, maternal infection, i.e., primary or recurrent, duration of rupture of membranes, integrity of mucocutaneous barriers, and mode of delivery, i.e., cesarean or normal.^10^

The incubation period varies from 4 to 21 days after delivery, with symptoms appearing between 6 and 21 days. The eruptions commonly involve the mouth, scalp, face, soles of the feet, and palms of the hand. Characteristically, vesicular eruptions appear as single unit or in clusters, measuring about 1 to 3 mm in diameter, which often ulcerate within a few days. The manifestations vary according to the type of infection (Flow Chart 2). Other constitutional symptoms include cyanosis and respiratory distress. Progressive symptoms include seizures, hepatitis, pneumonitis, and disseminated intravascular coagulation.^11^ Diagnostic modalities include serological tests, polymerase chain reaction amplification analysis of cerebrospinal fluid (CSF), and viral cultures. Treatment and prophylactic measures involve antiviral therapy with acyclovir.^10^ Prognosis of SEM infection is good (0% mortality rate), but 70% of infants with skin, eye, and mouth disease progress either to central nervous system or disseminated disease, both having a higher fatality or permanent sequelae.^11^

### Neonatal Candidiasis

Disseminated or invasive candidiasis is the second most common cause of mortality with a reported incidence of 2 to 20% in preterm newborns. The transmission of *Candida* can be vertical or due to external contaminations. The most common opportunistic *Candida* species include *Candida albicans* (75%) followed by *Candida glabrata, Candida krusei, Candida tropicalis,* and *Candida parapsilosis.* The risk factors comprise immature immune system, prolonged catheterization, prolonged hospital stay, etc. Oral manifestations include white plaques on oral mucosa, composed of hyphae, epithelial cells, and necrotic tissues. The prominent systemic features include meningitis, endophthalmitis, cardiovascular manifestations, and urinary tract infections.^12^ Diagnosis is confirmed by fungal cultures in blood, urine, and CSF. The colonization of oral mucosa by *Candida* organism plays a decisive role in development of invasive candidiasis; hence, as a prophylactic measure, maintenance of oral hygiene is believed to be an important preventive measure.^13^

**Flow Chart 2:**
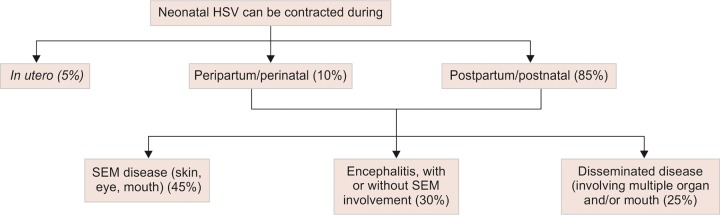
Types of neonatal herpes simplex infection

## TRAUMATIC LESIONS

### Mucocele

Mucocele commonly arises due to alterations in the minor salivary glands, occurring in approximately 2.7% of patients under the age of one.^14^ It can be of two types: Extravasation and retention mucoceles, the former affecting the lower lip most frequently. Extravasation type often affects the younger age group and results due to trauma.^14,15^ Clinically, it presents as bluish (depending on the proximity to the surface), translucent, and fluctuant swelling which may cause mechanical obstruction during feeding.^14^ Diagnosis can be confirmed by FNAB and histopathological evaluation. Conventional treatment includes surgical excision.^15^

### Ranula

Ranulas are a rarity in newborns, presenting as a swelling in the floor of the mouth and are commonly caused by extravasation of mucin than retention cyst. The incidence is estimated to be approximately 0.74%. Clinical features show marked similarity to that of a mucocele. Diagnosis can be arrived with the aid of FNAB, MRI, and histopathological assessment.^16^ Treatment includes observation for asymptomatic cases, aspiration, cryo-surgery, marsupialization, or surgical excision with or without sublingual gland depending upon the variant (cervical or plunging ranula).^17^

### Riga-Fede Disease

Riga-Fede disease (RFD) is a rare benign reactive mucosal disease first identified and later described by Riga (1881) and Fede (1890) respectively. The most contributory factor postulated is trauma. It is commonly associated with natal/neonatal teeth in newborns and other disorders like Riley-Day syndrome, Lesch-Nyhan syndrome, Tourette’s syndrome, and cerebral palsy. Dominguez-Cruz et al categorized RFD into “precocious RFD” (present within first 6 months of life, associated with natal-neonatal teeth, and has no correlation with neurological disorders) and “late RFD” (typically appearing after 6-8 months of life, associated with the first dentition, and may be related to neurological disorders).^18^ Clinically, the lesion appears ulcerated, unifocal/multifocal, and occasionally painful, frequently occurring on the ventral surface of tongue in the midline region. Other sites include lip, palate, gingiva, vestibular mucosa, and floor of the mouth. Diagnosis is confirmed by clinical examination and histopathology to rule out other possible cause of ulcerations caused by bacterial or fungal infections, immunologic diseases, and neoplasia. Treatment includes dental extraction, cortico-steroids, teething rings, oral disinfectants, smoothing the incisal edges, and use of protective dental appliances.^18-20^

### Breastfeeding Keratosis

Recently, Kiat-Amnuay and Bouquot reported a case of breastfeeding keratosis, nonresponsive to antifungal drugs, in a 2-month-old child. History elicited from parents revealed unusual habit of active lip sucking habit in-between the feeding sessions. Cytopathology revealed no mycotic structures. With diminution of habit, the lesion regressed by the 4th week with no recurrence.^21^

## AUTOIMMUNE DISEASES

### Neonatal Pemphigus Vulgaris

It is a rare vesiculobullous disease in neonates. Rucco et al first described the lesion in 1975 and till date more than 21 cases have been documented.^22^ It is caused due to transplacental passage of maternal immunoglobulin G autoantibodies (mainly class 4) against transmembrane glycoprotein desmoglein 3 (Dsg3). It is characterized by multiple cutaneous, mucosal, or mucocutaneous ulcerations soon after birth. Diagnosis is confirmed by histopathology and immunofluorescence. The symptoms spontaneously resolve within 2 to 3 weeks.^22^

## TUMORS

### Hemangioma

Hemangiomas are most common pediatric vascular benign neoplasms with 2 to 3 and 22 to 30% prevalence in neonates and underweight preterm newborns, respectively, with a female predilection (3:1-5:1). It commonly involves the head and neck region (60%) apart from trunk (25%) and extremities (15%). Common oral locations include lips, buccal mucosa, tongue, and rarely the palate and uvula.^23^ Various predisposing factors include child-bearing age, gestational hypertension, and infant birth weight. Kasabach-Merritt syndrome has been associated with extensive hemangiomas in infants.^24^ They are usually present at birth but tend to develop few weeks after. The natural course of hemangioma follows a rapid proliferating phase (0-1 year), involuting phase (1-5 years), and involuted phase (5-10 years). Clinically, it manifests as rapidly growing macule followed by regression into spotted pigments. Diagnosis is confirmed by history, FNAC, MRI, and/color Doppler USG, histopathology, and immunohistochemistry (Glucose transporter 1) ruling out other vascular malformations.^25^ Treatment guidelines are based on the stage of the lesion and includes drugs (pro-pranolol, corticosteroids, a-interferon), lasers (CO_2_, diode, flash lamp pulsed dye), and surgical corrections. Complete resolution occurs in 70% cases but around 40 to 50% of the cases show permanent changes in the skin, such as telangi-ectases, stippled scarring, anetoderma, hypopigmentation, fibro-fatty residua, etc., without any disfigurement.^26^

### Lymphangioma

Lymphangiomas are benign neoplasms of the lymphatic channels with 50% of cases noted at birth and 90% developing before the age of 2. The prevalence is 1-3/10,000 live births, affecting both the genders equally, involving 75% of the head and neck region followed by trunk, abdomen, and extremities.^24,27^ Incomplete development; ectopic deposition of lymph tissues; congenital obstruction or sequestration of the primitive lymphatic channels; and role of vascular endothelial growth factor (VEGFR)-2 and 3 are suggested modes of etiopathogenesis. Histologically, it can be categorized into simplex form, cavernous, cystic and benign lymphangioendothelioma. Oral sites include the dorsum of tongue, lips, buccal mucosa, soft palate, and floor of the mouth in the descending order of preference. Clinically, it is a slow, progressive lesion with superficial blue-black or red hemorrhagic elevated nodules, the deeper lesions presenting as soft, diffuse growths with normal color and cystic forms manifesting as soft, painless fluid-filled lesions, characteristically located in the neck. Oral manifestations include macroglossia, sialorrhea, dysphagia, cosmetic abnormalities, ulcerations, deformity of jaws, and difficulty in speech and mastication and feeding problems. Other features include respiratory distress, infection and fever, and sudden exaggeration. Diagnosis is confirmed by clinical examination, MRI/CT/color Doppler USG, and histopathology as well as immunohistochem-istry lymphatic markers, such as D2-40, Prox-1, factor VIII-associated antigen, CD-31, LYVE-1, podoplanin, and VEGFR-3. Prenatal diagnosis can be aided by chromosomal analysis of chromosomes 13, 18, 21, X, and Y. Treatment modalities include surgery, cryotherapy, electrocautery, sclerotherapy, steroids, embolization, and ligation, laser surgery (neodymium-doped yttrium aluminum garnet, CO_2_), radiofrequency tissue ablation technique, and radiation therapy. Recurrence is high, 39% in case of tongue, followed by hypopharynx and larynx.^27^

### Langerhans’ Cell Histiocytosis X

Langerhans cell histiocytosis X (LCH) constitutes a wide group of disorders, sharing the histiocyte as the common cell of origin. Langerhans cell histiocytosis X occurs at a frequency of 1 in 200,000 children under 15 years with a male predilection. It is classified as eosinophilic granu-loma, Hand-Schuller-Christian disease (between 3 and 6 years of age), and Letterer-Siwe disease (under 2 years of age).^28^ Another variant, congenital self-healing reticu-lohistiocytosis/Hashimoto-Pritzker, is present at birth and shows complete involution within 2 to 3 months. Characteristic features include erythematous vesiculo-pustules with/without crusting and eczematous scaling with respect to skin.^29^ Oral manifestations include pete-chiae, lytic bone lesions involving posterior mandible, and pain and swelling of gingiva corresponding with accumulation of Langerhans cells. Other sites presenting with lytic lesions include skull, femur, pelvis, and vertebrae. Systemic findings may include hepatomegaly, splenomegaly, pulmonary affection, pancytopenia, central nervous system involvement, diabetes insipidus, etc., depending upon the severity. Diagnosis is confirmed by histopathology and immunohistochemistry (CD1a and/or S100) and complete liver and blood chemistry profile with CT/MRI to evaluate the extent of systemic involvement. Possible differentials include seborrheic dermatitis, lytic lesions of jaws, leukemia, lymphoma, and metastatic tumors.^28^ Treatment modalities include surgical intervention, chemotherapy, and radiotherapy. Prognosis depends upon the age and extent of systemic involvement.^28,29^

### Congenital Epulis of Newborn

It is one of the rare benign tumors of oral cavity first described by Neumann in 1871.^30,31^ The tumor shows a female predilection (8:1-10:1 ratio) with an estimated incidence of 0.0006%.^32^ The various etiology proposed includes odontogenic, neurogenic, myoblastic, endocrino-logic, fibroblastic, or histiocytic origin.^31^ The lesion occurs three times more commonly in the maxilla than mandible, frequently involving the alveolar ridge near the canine region. Clinical presentation includes lobular or ovoid, sessile or pedunculated swelling of various sizes, covered by a smooth normal/reddish mucosal surface. Larger lesions cause mechanical obstruction in feeding and respiration in neonates. Diagnosis is confirmed by USG, CT/MRI, and histopathology, which appears similar to other granular cell tumors in adults, but is distinguished based on its exclusive origin from the neonatal gingiva, the scattered presence of odontogenic epithelium, lack of interstitial cells with angulate bodies, and the more elaborate vasculature.^30^ Other possible differential diagnosis includes dermoid cysts, teratoma, hemangioma, lymphatic malformations, rhabdomyosarcoma, and melanotic neuroectodermal tumors of infancy (MNTI). Treatment includes complete surgical excision with no reported cases of recurrence.^31^

### Melanotic Neuroectodermal Tumor of Infancy

Melanotic neuroectodermal tumor of infancy is a rare pigmented benign neoplasm. It is seen in the first 6 months of life with a male predilection. It originates from the neural crest cells. It commonly presents in the craniofacial region (92.8%), maxilla (61.4%), skull (15.7%), mandible (6.4%), the brain (5.7%), and the genitals. Oral sites include tongue, palate, buccal mucosa, and floor of the mouth. Clinically, it is characterized by painless, nonulcerative, expansile, rapidly growing, pigmented, and locally aggressive behavior. Diagnosis is based on clinical assessment, CT/MRI, and histopathology. Microscopically, it presents as a biphasic tumor, comprising neuroblast-like round cells and melanocytic cells. Differential diagnosis includes Ewing’s sarcoma, desmoplastic small round cell tumor, rhabdomyosar-coma, peripheral neuroepithelioma, neuroblastoma, peripheral primitive neuroectodermal tumor, lym-phoma, and malignant melanoma. Treatment modalities include surgical excision, chemotherapy, and radiotherapy alone or combined. High recurrence (10-15%), metastases (3%), and malignant transformation rate (6.5%) are reported.^33,34^

### Epignathus

Epignathus or pharyngeal teratoma is an exceptionally rare form of teratoma attached to the base of the skull, usually the hard palate or mandible. It frequently occurs in females (3:1 ratio) with an incidence of approximately 1 in 35,000 to 200,000 live births. Teratomas are derived from the pleuripotent cells of one or more of the three germ layers. It presents as a unidirectional growth protruding through the oral cavity with rare intracranial extension and in association with cleft palate and bifid tongue or nose. Other manifestations include elevated maternal serum alpha-fetoprotein, mechanical feeding, airway obstruction, and fetal death. Diagnosis is confirmed with clinical examination, USG, and CT/MRI excluding hemangioma, lymphangioma, dermoid cyst, sincipital encephalocele and other benign or malignant soft-tissue masses of neonates. Prognosis is poor and mortality rate is high in both fetal life and neonates.^35^

### Oral Choristoma

Choristomas are histologically normal tissue, presenting in its nonnative location. Oral choristomas are exception-ally rare aberrant developmental disorders with a male predilection. In the head and neck region, the most frequent locations include the tongue (lingual choristoma), floor of mouth, pharynx, and hypopharynx.^36^ Oral choris-toma can include salivary gland, cartilaginous, osseous, lingual thyroid, sebaceous, glial, gastric, and respiratory mucosa.^37^ Clinically, it presents as asymptomatic, large masses, causing obstruction in feeding and respiration in neonates. Diagnosis is aided by clinical examination, imaging, and histopathology, ruling out other similar oral neonatal lesions like mucocele, lymphatic malformation, venous malformation, and dermoid cyst. Treatment includes complete surgical excision. No recurrence is reported.^36^

### Sialoblastoma

Sialoblastoma was coined by Taylor (1988).^39^ It is a rare salivary gland neoplasm of epithelial origin, with approximately 30 reported cases. The neoplasm resembles the developmental phase of the salivary glands. It is noted as a swelling of variable sizes involving the parotid followed by the submandibular gland. Facial nerve palsy may be present in cases of parotid gland involvement. Treatment includes surgical excision. Local recurrence is reported. Chemotherapy and radiotherapy are contraindicated. Prognosis is favorable.^38,39^

### Hemangioendothelioma (HAE) of the Parotid Gland

It is benign neoplasm of the salivary gland, present at birth but manifests later. It commonly affects females (3:1). Its natural course includes the proliferative followed by involution phase. Clinically, it manifests as multiple, rapidly growing parotid mass, often along with the presence of cutaneous lesion (infantile hemangioma). Diagnosis is aided by imaging and histopathology. Other newborn lesions involving the parotid gland include sialoblastoma, congenital infantile fibrosarcoma, solitary infantile myofibromatosis, and vascular malformations. Surgical resection carries risk of facial nerve damage.^40^

### Malignant Neoplasms

In a 40-year review of solid malignant neoplasm in neonates by Hasen Xue et al (1995) it was revealed that malignant neoplasms are extremely rare in newborns and involvement of oral tissues even rarer. The majority of cases involved head and neck followed by trunk and extremities. Oral cavity involvement was seen in only two cases: Malignant melanoma (hard palate) and spindle cell sarcoma (tongue).^41^ In another review of 60 years by Campbell et al (1987) only one case of rhabdomyosarcoma (tongue) was observed. Such cases require prompt diagnosis and multimodal management for a disease-free survival.^42^

## EFFECT OF PRETERM BIRTH IN ORAL TISSUES

The most common cause of neonatal deaths is often associated with preterm birth and its related complications. In brief, the effects of premature birth on oral tissues mainly include dilacerations of crown from endotracheal intubation, distortions of dental arches, enamel opacity, and enamel hypoplasia, increase in height of the palate, delay in eruption of the primary dentition, and growth of the permanent dentition.^43^

## CONCLUSION

Neonates presenting with intraoral lesions mandate precise diagnosis, management, and parental reassurances and counseling. A thorough evaluation and substantial knowledge can aid in diagnosis of easily recognizable as well as rare abnormalities affecting the oral tissues in neonates.
